# Supplementary material for: MetaRibo-Seq measures translation in microbiomes
Source: Nat Commun. 2020 Jun 29;11:3268. doi: 10.1038/s41467-020-17081-z (PMC7324362; doi:10.1038/s41467-020-17081-z)
Supplement: Supplementary file 10 — Supplementary Data 7 [file 41467_2020_17081_MOESM10_ESM.zip › File2/Confidence_High_Taxonomy/64580_out.krona.html]

Javascript must be enabled to view this page.

members
magnitude
magnitudeUnassigned
count
unassigned
taxon
rank

64580\_out

2

2
2
superkingdom

phylum
976
2

200643
class
2

171549
order
2

2
815
family

2
816
genus

1

SRS018888\_contig\_number\_10226
817
species

1

SRS144506\_contig\_number\_51633
species
28116
